# Supplementary material for: Heart rate recovery in 1 minute after the 6-minute walk test predicts adverse outcomes in pulmonary arterial hypertension
Source: PLoS One. 2022 May 27;17(5):e0268839. doi: 10.1371/journal.pone.0268839 (PMC9140226; doi:10.1371/journal.pone.0268839)
Supplement: Attachment — Submitted filename: Response to Reviewers.docx [file pone.0268839.s002.docx]

Dear Editor-in-Chief and Reviewers,

We would like to thank all of you for the suggestions you’ve made about our paper. We reviewed the text and answered topic by topic as below. All the modifications made are highlighted on the text. We are very grateful that all the suggestions brought great improvement to the article.

Kindest regards,

The Authors.

**Reviewer 1:**

1. The Methods section needs a description of inclusion/exclusion criteria, the study institutional approval number/information, and details on obtaining informed consent and protected management of human subject data.

A - We agreed and did the changeas suggested.

2. The authors might consider increasing the data on the cohort. Such details as BMI, associated medical conditions, tobacco usage, cause of death, etc. would be of interest.

A - We agreed and added BMI, comorbidities, previous tobacco usage as suggested in table 1.

In page 9, line 182, we added the causes of death.

3. The authors might put together a second table that summarizes other studies of similar design and intent. The reader would find this a helpful addition to the author's original work and could be mentioned in the Discussion section.

A - We agreed and included table 2 in the discussion section.

4. Table 1 may have a few abbreviations not spelled out in the legend. Please correct.

A - We corrected the legend.

5. The figures are quite blurry and should be sharped up and improved.

A – We improved the figures and uploaded the files to the Preflight Analysis and Conversion Engine (PACE) digital diagnostic tool.

**Reviewer 2 :**

1.  I suggest that the authors complete Table 1 with the medications used, the number of patients who used oxygen, how many stopped during the six-minute walk test, or if others also stopped. Adding how many desaturate during the test, this is very relevant. I suggest adding the percentage of the predicted distance traveled for the population studied.

A - We agreed and added medications used at the time of the six-minute wak test (diuretic and oxygen), how many stopped and desaturate during this test and the percentage of the predicted distance calculated by the reference equation in the Brazilian population as suggested in table 1. For this reason, we added the reference 14.

Best,

The authors
